# Pain Input After Spinal Cord Injury (SCI) Undermines Long-Term Recovery and Engages Signal Pathways That Promote Cell Death

**DOI:** 10.3389/fnsys.2018.00027

**Published:** 2018-06-21

**Authors:** Joel D. Turtle, Misty M. Strain, Joshua A. Reynolds, Yung-Jen Huang, Kuan H. Lee, Melissa K. Henwood, Sandra M. Garraway, James W. Grau

**Affiliations:** ^1^Lab of Dr. James Grau, Department of Psychology, Cellular and Behavioral Neuroscience, Texas A&M University, College Station, TX, United States; ^2^United States Army Institute of Surgical Research, JBSA-Fort Sam Houston, San Antonio, TX, United States; ^3^Department of Physiology, Emory University School of Medicine, Atlanta, GA, United States

**Keywords:** spinal cord injury, pain, capsaicin, cell death, apoptosis, pyroptosis, recovery

## Abstract

Pain (nociceptive) input caudal to a spinal contusion injury increases tissue loss and impairs long-term recovery. It was hypothesized that noxious stimulation has this effect because it engages unmyelinated pain (C) fibers that produce a state of over-excitation in central pathways. The present article explored this issue by assessing the effect of capsaicin, which activates C-fibers that express the transient receptor potential vanilloid receptor-1 (TRPV1). Rats received a lower thoracic (T11) contusion injury and capsaicin was applied to one hind paw the next day. For comparison, other animals received noxious electrical stimulation at an intensity that engages C fibers. Both forms of stimulation elicited similar levels of *c-fos* mRNA expression, a cellular marker of nociceptive activation, and impaired long-term behavioral recovery. Cellular assays were then performed to compare the acute effect of shock and capsaicin treatment. Both forms of noxious stimulation increased expression of tumor necrosis factor (TNF) and caspase-3, which promotes apoptotic cell death. Shock, but not capsaicin, enhanced expression of signals related to pyroptotic cell death [caspase-1, inteleukin-1 beta (IL-1ß)]. Pyroptosis has been linked to the activation of the P2X7 receptor and the outward flow of adenosine triphosphate (ATP) through the pannexin-1 channel. Blocking the P2X7 receptor with Brilliant Blue G (BBG) reduced the expression of signals related to pyroptotic cell death in contused rats that had received shock. Blocking the pannexin-1 channel with probenecid paradoxically had the opposite effect. BBG enhanced long-term recovery and lowered reactivity to mechanical stimulation applied to the girdle region (an index of chronic pain), but did not block the adverse effect of nociceptive stimulation. The results suggest that C-fiber input after injury impairs long-term recovery and that this effect may arise because it induces apoptotic cell death.

## Introduction

Spinal cord injury (SCI) represents a significant cost to society, both in the direct costs of treating patients and even greater indirect costs including loss of employment and caregiver time (DeVivo, 1997). Spinal cord injuries disproportionately impact the young and active, particularly athletes and soldiers (Wyndaele and Wyndaele, 2006). The vast majority of SCI cases occur following traumatic accidents and are often accompanied by associated injuries (e.g., fractures, lacerations and abrasions). These associated injuries could provide a continued source of C-fiber input throughout recovery that may significantly impact outcomes.

Prior work has shown that noxious stimulation can impact spinal cord function and undermine neural plasticity (Grau et al., 1998; Crown et al., 2002; Joynes et al., 2003; reviewed in Grau et al., 2006, 2012, 2017). These findings emerged from studies examining whether neurons caudal to a complete spinal cord transection could support learning (Grau et al., 1998; Grau, 2014; Joynes and Grau, 2004). For this purpose, brief (80–100 ms) electrical stimulation (shock) was used to engage peripheral sensory fibers. Intermittent stimulation applied on a variable (0.2–3.8 s) schedule, in an uncontrollable manner, sensitized behavioral reactivity to mechanical stimulation and inhibited adaptive learning. Just 6 min of intermittent stimulation undermined learning for 24–48 h. The development and expression of this learning deficit depends upon signaling through the pro-inflammatory pathways of tumor necrosis factor-α (TNF-α) and interleukin-1β (IL-1β; Young et al., 2007; Vichaya et al., 2009). It was hypothesized that the induction of this effect was linked to the activation of C-fibers and the sensitization of nociceptive pathways (Ferguson et al., 2006, 2012). Supporting this, electrical stimulation of the sciatic nerve only affected adaptive plasticity when given at intensities sufficient to engage C-fiber activity (Baumbauer et al., 2008). More importantly, selectively engaging C-fibers that express the transient receptor potential vanilloid receptor-1 (TRPV1) with the TRPV1 agonist capsaicin increased neural excitability (as evidenced by the expression of the immediate early proto-oncogene *c-fos*, Huang et al., 2016) and impaired adaptive learning for 24 h (Hook et al., 2008).

To explore whether pain input affects recovery after SCI, noxious electrical stimulation was applied a day after rats received a moderate contusion injury (Grau et al., 2004). Variable intermittent shock applied to the tail or leg in an uncontrollable manner impaired the recovery of locomotor function. Across experiments, we found that shock increased the incidence of spasticity, increased tissue loss at the site of injury, slowed the recovery of bladder function, and promoted the development of chronic pain (Grau et al., 2004). Cellular assays showed that the adverse effects of shock are linked to increased expression of TNF, cellular excitation (*c-fos* mRNA), and the activation of signal pathways (e.g., caspase 3) that are implicated in apoptotic cell death (Garraway et al., 2011, 2014). Immunohistochemistry revealed that *c-Fos* protein was co-localized with a neuronal marker (NeuN) whereas caspase-3 was co-localized with both OX-42 (microglia) and NeuN, but not GFAP (astrocytes; Garraway et al., 2014).

Conclusions from past work are limited by the use of shock to engage sensory fibers. The advantage of this form of stimulation is that intensity and duration are readily controlled. The disadvantage is that intense electrical stimulation engages a broad range of myelinated and unmyelinated sensory fibers. For this reason, it remains unclear whether activation of C-fibers alone is both necessary and sufficient to induce these adverse effects. Having previously shown that electrical stimulation at an intensity that engages C-fibers is required, the current study addresses the issue of sufficiency using the irritant capsaicin, which selectively activates TRPV1 expressing C-fibers (Willis, 2001). Here we tested whether capsaicin treatment impairs long-term recovery and acutely engages signal pathways associated with apoptotic cell death. As a positive control, we also examined the effect of shock.

A second limitation of past work is that we have only explored the impact of noxious stimulation on one form of cell death (apoptosis). Recent work has shown that SCI can also engage a form of pro-inflammatory cell death known as pyroptosis (de Rivero Vaccari et al., 2008, 2016; Mortezaee et al., 2018). Pyroptosis was originally discovered in circulating macrophages and is defined by the activation of caspase-1 (Fink and Cookson, 2006; Sharma and Kanneganti, 2016). This protease is responsible for the processing of the pro-inflammatory cytokines interleukin-1β (IL-1β) and interleukin-18 (IL-18) from their immature forms into their mature, biologically active forms (Cerretti et al., 1992; Wilson et al., 1994). In addition to its role in processing IL-1β and IL-18, caspase-1 is known to degrade a number of other targets and promote cell death (Shao et al., 2007; Denes et al., 2012). Therapeutics targeting caspase-1 activation and pyroptosis have been shown to improve functional recovery and histopathological scores in an animal model of SCI (de Rivero Vaccari et al., 2008, 2016). To evaluate whether this form of cell death is amplified by noxious stimulation, we evaluated the expression of caspase-1, IL-1β and IL-18 (Galluzzi et al., 2012). Our results imply that the relative contribution of apoptosis vs. pyroptosis depends upon the form of nociceptive stimulation; only shock strongly engaged signal pathways related to pyroptosis.

The activation of caspase-1 has been linked to pathological purinergic signaling (Bernier, 2012) involving the P2X7 receptor, which regulates the flow of adenosine triphosphate (ATP) through the pannexin-1 channel. In pyroptosis, the outward flow of ATP through the pannexin-1 channel can engage the P2X7 receptor, inducing a form of positive feedback that fuels further ATP release and the activation of caspase-1 (Wicki-Stordeur and Swayne, 2014). We tested whether blocking the P2X7 receptor with Brilliant Blue G (BBG), or the pannexin-1 channel with probenecid, inhibits the activation of caspase-1 and IL-1ß. Reasoning that blocking both the P2X7 receptor and the pannexin-1 channel could have an additive effect, we also assessed their combined effect. In injured rats that had received shock, only BBG inhibited the activation of signals related to pyroptosis. BBG promoted long-term recovery and attenuated a marker for chronic pain, but did not attenuate the adverse effect of nociceptive stimulation.

## Materials and Methods

### Animals

Adult male Sprague-Dawley rats (100–120 days old) were obtained from Envigo (Houston, TX, USA) and acclimated for at least 7 days prior to experimentation. Before contusion, animals were dual housed with water and food *ad libitum* and maintained on a 12-h light-dark cycle. Behavioral testing and surgeries were performed during the light portion of the cycle. All experiments were carried out in accordance with NIH standards for the care and use of laboratory animals (NIH publication No. 80-23), and were approved by the Institutional Animal Care and Use Committee at Texas A&M University. Every effort was made to minimize suffering and limit the number of animals used.

### Spinal Contusion

All rats received a moderate contusion injury at the T10-11 vertebral level using the MASCIS device. Anesthesia was induced using a mixture of 5% isoflurane in medical oxygen and maintained at a concentration of 2%–3% during surgery. Two longitudinal incisions were made on either side of the vertebral column extending approximately 2 cm rostral and caudal to the injury site. The T10-11 vertebrae were located by palpation, exposed and a laminectomy was performed. The dura remained intact. The MASCIS device was then secured around the vertebral column and the 10-g impactor centered on the lesion site. The drop height was set at 12.5 cm. After surgery, the wound was closed using Michel clips. To prevent urinary tract infection and compensate for fluid loss, subjects received 1,00,000 units/kg of penicillin and 3 mL of saline after surgery.

After surgery, animals were singly housed and allowed to recover overnight (18–24 h) in a temperature-controlled room (25°C) with water and food *ad libitum*. Subjects were transferred back to standard housing on the first day after injury.

### Drug Preparation

BBG and probenecid drug treatments were prepared in 1 mL of sterile phosphate buffered saline (PBS) at pH 7.4. BBG was dissolved directly in PBS at the required concentrations. Probenecid was dissolved in a small volume of 1 M sodium hydroxide (NaOH). Then, PBS was added and the pH adjusted to 7.4 using monobasic potassium phosphate. The drug combination was created by preparing BBG and probenecid solutions at twice the required concentrations, then mixing the two solutions in equal parts. BBG and probenecid drug treatments (100 mg/kg) were administered 3, 12 and 24 h following injury. All drugs were given by intraperitoneal (i.p.) injection.

### Noxious Stimulation

Noxious electrical stimulation was applied while animals were loosely restrained in opaque Plexiglas tubes housed in an acoustic isolation chamber. Electrical stimulation was applied through tail electrodes formed from a modified fuse clip, as previously described (Grau et al., 1998). Briefly, the electrodes were coated with electrode gel (Harvard Apparatus, Holliston, MA, USA) and attached 2 cm from the tip of the tail with Orthaletic tape. The electrodes were attached to a BRS/LVE shock generator (Model SG-903), and constant current 1.5-mA, AC (60 Hz) electrical stimuli (100 ms in duration) were applied on a variable intermittent schedule (0.2–3.8 s; rectangular distribution) for 6 min. Unshocked controls were treated the same except shock was withheld.

The irritant capsaicin was also applied while animals were restrained in Plexiglas tubes, modified to allow access to each hind limb (see Grau et al., 1998). Animals received a 50 μL intradermal injection of 3% capsaicin on the dorsal side of one hind paw using a 27-gaugage needle. Controls received the same volume of vehicle (7% Tween-20). Whether the left or right paw was injected was counter-balanced across animals. The animals remained in the apparatus for a total of 6 min, and then were returned to their home cage.

### Assessment of Recovery

Health checks were performed daily throughout the recovery period. Animals were examined for signs of autophagy, stress and infection. Weight was assessed daily as a measure of general health. Bladders were expressed manually twice per day until voluntary control was established (six consecutive expressions with no urine).

Locomotor function was assessed using the scoring system developed by Basso, Beattie and Bresnahan (BBB; Basso et al., 1995) while animals explored an open field. Locomotor function was assigned 1 day after injury prior to noxious stimulation (Pre). Animals were then assigned to treatment conditions in a manner that ensured that injury severity was balanced across groups. To assess the long-term effects of noxious stimulation on locomotor performance, animals were tested every day during the first week on days 10 and 14, and weekly thereafter. Care was taken to assure that individuals performing behavioral scoring had high inter-observer reliability (>95%) and were unaware of the animals treatment condition.

### Reactivity to Mechanical and Thermal Stimulation

Reactivity to mechanical stimulation applied to the plantar surface of each hind paw was tested using von Frey filaments (Stoelting, Wood Dale, IL, USA). Animals were tested while loosely restrained with the hind limbs hanging freely below a Plexiglas tube. After a 15 min acclimation period, the filament series was tested using the up-down technique (Chaplan et al., 1994), recording the filament thickness that elicited a flexion response. At-level pain was determined by counting the number of vocal responses to a 26-g mechanical stimulus applied on a four by eleven grid across the girdle region of the rat. Reactivity to a thermal stimulus was assessed using the IITC Tail Flick Analgesia Meter. Again, animals were loosely restrained in Plexlglas tubes. The thermal stimulus was applied approximately 3 cm from the tip of the tail and terminated after 8 s to avoid tissue damage.

### Tissue Collection and Cellular Assays

Animals were euthanized with 100 mg/kg of pentobarbital and 1 cm of spinal cord tissue centered at the lesion was dissected and flash frozen in liquid nitrogen. Tissue was prepared and mRNA/protein levels were assessed as described in Garraway et al. (2011, 2014). Briefly, the cord was processed for the extraction of both total RNA (RNeasy Mini Kit; Qiagen, Valencia, CA, USA) and total protein (see later in the text). Total RNA (100 ng) was converted into cDNA using TaqMan EZ RT-PCR Core reagents (Applied Biosystems, Carlsbad, CA, USA), and the mRNA levels of all targets were measured by TaqMan quantitative real-time (RT)-PCR using a StepOnePlus™ Real-Time PCR System (Applied Biosystems, Carlsbad, CA, USA), with ß-Actin serving as a control gene. The probes and primers for ß-actin and *c-fos* were obtained from Applied Biosystems.

After RNA extraction, total protein was extracted from the organic layer using the QIAzol lysis reagent protocol for isolation of genomic DNA and/or proteins from fatty tissue (Qiagen, Valencia, CA, USA). A Bradford assay (BioRad, Hercules, CA, USA) was used to determine the concentration of protein extracts. Protein samples were diluted in 4× Laemmli buffer to a final concentration of 3 mg/mL. Western blot analysis was then used to quantify TNF, caspase-1, 3 and 8, IL-1ß and IL-18. After transfer onto PVDF membranes (Millipore, Bedford, MA, USA), the blots were blocked for 1 h in 5% blotting-grade milk (BioRad, Hercules, CA, USA) in Tris-buffered saline Tween-20 (TBST). After blocking, the blots were incubated overnight at 4°C in one of the following primary antibodies generated in rabbit: TNF alpha (1:500; #ARC3012—Invitrogen, Camarillo, CA, USA; AB_305641), caspase-1 (1:1000; #ab1872—Abcam, Cambridge, MA, USA; AB_302644), caspase-3 (1:1500; #NB600-1235—Novus Biological, Littleton, CO, USA; AB_2069897), caspase-8 (1:1000; #NB100-56116—Novus Biological, Littleton, CO, USA; AB_837874), IL-1ß (1:200; #sc-7884—Santa Cruz Biotechnology, Santa Cruz, CA, USA; AB_2124476), IL-18 (1:200; #sc-7954—Santa Cruz Biotechnology, Santa Cruz, CA, USA; AB_1564060), or lamina B (1:1000; #ab16048—Abcam, Cambridge, MA, USA; AB_443298). The next day, blots were washed in TBST (3 × 10 min) at room temperature then incubated in HRP-conjugated goat anti-rabbit secondary antibodies (1:5000; #31460; Pierce, Rockford, IL, USA) for 1 h at room temperature. After another 3 × 10 min series of washes, the blots were developed with electrochemiluminescence (Pierce, Rockford, IL, USA) and imaged with Fluorchem HD2 (ProteinSimple, Santa Clara, CA, USA). Ratios of the integrated densitometry of each protein of interest to the loading control (lamina B) were calculated and normalized to a control group (run on the same blot) that did not receive nociceptive stimulation.

### Experimental Designs

The effect of nociceptive stimulation on mRNA and protein expression was assessed in rats that had undergone a moderate contusion injury. A day after injury, locomotor performance was assessed and noxious stimulation was applied as described above. A quarter of the animals were treated with capsaicin while another quarter served as the vehicle treated controls. Likewise, one fourth of the rats received shock while the remaining animals served as the unshocked controls. Tissue was collected 1, 3, or 24 h after nociceptive stimulation. This yielded a 2 [noxious stimulation (or none)] × 2 [pain type (via peripheral injection or shock electrodes)] × 3 [time of tissue collection (1, 3, or 24 h)] experimental design with six animals per treatment condition.

A similar procedure was used in a separate experiment to prepare animals for the assessment of long-term recovery, using a 2 (noxious stimulation) × 2 (pain type) factorial design with six rats per condition. A day after injury, locomotor performance was assessed and rats were treated with capsaicin, vehicle, shock, or nothing (unshocked). Locomotor performance was assessed over the next 4 weeks as described above. At the end of the recovery period, tissue at the site of injury was prepared for protein assays.

To evaluate the effect of drug treatment on nociception-induced protein expression, locomotor performance was scored 2 h after injury. Half of the rats then received BBG (100 mg/kg), probenecid (100 mg/kg), or BBG + probenecid via an i.p. injection. These doses were chosen based on previously published results (Peng et al., 2009; Adamczak et al., 2014). For each drug treatment, an equal number of animals received a vehicle injection. Animals received the first i.p injection 3 h after injury and two additional injections at 12 and 24 h after injury. Thirty minutes after the last injection, rats were placed in the restraining tubes and had shock electrodes attached to the tail. Half the rats in each drug condition then received shock while the remaining animals served as the unshocked controls. This yielded a 2 (BBG or vehicle) × 2 (probenecid or vehicle) × 2 [noxious stimulation (shock or unshocked)] experimental design with six animals per treatment condition. Tissue was collected 3 h after shock treatment and prepared for Western blotting as described above.

The fourth experiment assessed whether BBG treatment affects the long-term consequences of nociceptive stimulation. Again, locomotor performance was scored 2 h after injury. Half the animals then received three i.p. injections of BBG (100 mg/kg) at 3, 12 and 24 h after injury. The remaining animals received the vehicle. Thirty minutes after the last injection, rats were placed in the restraining tubes and half the animals in each drug condition were given shock while the remaining rats received nothing (unshocked). This yielded a 2 [drug treatment (BBG or vehicle)] × 2 [noxious stimulation (shock or nothing)] factorial design with six animals per treatment condition. Locomotor performance and weight were monitored for 6 weeks. At the end of the recovery period, behavioral reactivity to mechanical stimulation applied to the paw or girdle region, and responsiveness to a thermal stimulus applied to the tail (tail-flick test), was assessed as described above.

### Statistics

All of the experiments employed full factorial designs and an equal number of animals per condition. Prior work has shown that the experimental treatments examined have a large effect size (*d* > 1.4). A power analysis confirmed that our sample size (6/group) was sufficient to achieve statistical significance with this effect size. Animals were randomly assigned to experimental treatments and the researchers conducting behavioral or cellular assays were blind to treatment condition. All data were analyzed using analysis of variance (ANOVA) or analysis of covariance (ANCOVA). When necessary, *post hoc* comparisons of the group means were performed using Duncan’s New Multiple Range test. In all cases, a criterion of *p* < 0.05 was set as the threshold for statistical significance.

## Results

### Noxious Stimulation Increases *c-Fos* mRNA Expression

**Figure 1:**
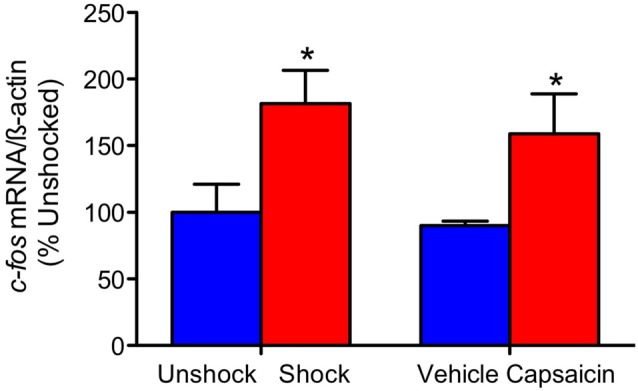
*c-fos* mRNA expression. Exposure to shock or capsaicin a day after a moderate contusion increased *c-fos* mRNA expression at the site of injury (*n* = 6). Error bars represent the standard error of the mean (SEM). *p < 0.05.

Our comparison of shock and capsaicin treatment assumes that both forms of stimulation engage a comparable level of nociceptive activity. To explore this issue, we assessed the expression of the immediate early gene *c-fos*. This target was selected because it is rapidly engaged by noxious stimulation, is widely used as an index of nociceptive activity within the pain literature, and is typically engaged by treatments that produce EMR and nociceptive sensitization (Willis, 2001; Ferguson et al., 2006, 2012; Huang et al., 2016). Here, we explored whether these treatments exert similar cellular effects at the site of injury. Contused rats were administered shock or treated with capsaicin a day after injury. An equal number of animals received nothing (unshocked) or an injection of the capsaicin vehicle (vehicle). Three hours later a 1-cm segment of the spinal cord, encompassing the injury site, was collected and prepared for real-time RT-PCR. Noxious stimulation induced an increase in *c-fos* expression at the site of injury (Figure 1), *F*_(1,20)_ = 9.57, *p* = 0.0057, and the magnitude of this effect did not vary across pain type, *F*_(1,20)_ < 1.0, *p* > 0.05.

### Both Capsaicin and Shock Treatment Impair Long-Term Recovery

**Figure 2:**
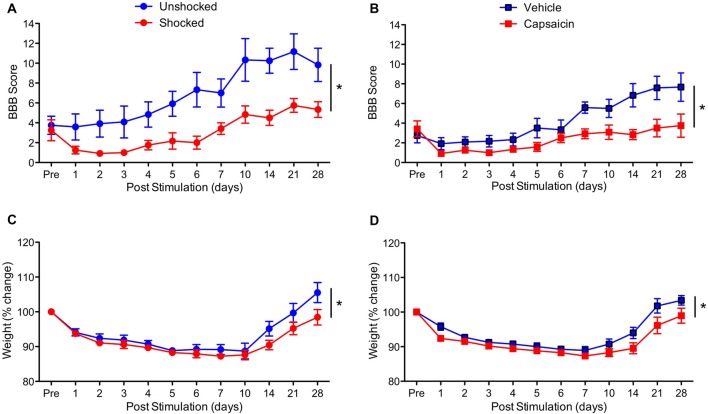
Impact of shock or capsaicin treatment on long-term recovery. **(A)** Rats exposed to variable intermittent shock (Shocked) a day after injury exhibited poor locomotor recovery as indexed by the scale developed by Basso, Beattie and Bresnahan (BBB Score; Basso et al., 1995). **(B)** Exposure to capsaicin a day after injury undermined long-term locomotor recovery. Exposure to shock **(C)** or capsaicin **(D)** also slowed the recovery of weight. Error bars indicate the SEM (*n* = 6). *p < 0.05.

To determine whether treatment with a peripheral irritant impairs long-term recovery, contused rats were treated with capsaicin or its vehicle a day after injury. As a positive control, an equal number of rats received shock or remained unshocked. Behavioral recovery was monitored over the next 28 days using the BBB locomotor score (Basso et al., 1995). As expected, vehicle treated rats and the unshocked controls recovered some locomotor function (Figures 2A,B). Relative to these controls, both shock and capsaicin impaired long-term recovery. In addition, it appears that rats that received a peripheral injection generally exhibited poorer recovery. To control for variation in injury severity, the data were analyzed using an ANCOVA with the pre-stimulation BBB score serving as the covariate. Pain treatment impaired long-term recovery, *F*_(1,19)_ = 10.64, *p* = 0.0041, and the magnitude of this effect did not vary across pain type, *F*_(1,19)_ = 1.41, *p* > 0.05. The within subjects terms revealed a significant effect of recovery day, *F*_(10,190)_ = 32.66, *p* < 0.0001, and that the magnitude of change observed across days differed depending upon whether rats had received noxious stimulation, *F*_(10,190)_ = 6.17, *p* < 0.0001. The inferior recovery observed in injected animals yielded a Pain Type × Recovery Day interaction, *F*_(10,190)_ = 2.97, *p* = 0.0017. Importantly, both forms of noxious stimulation disrupted locomotor recovery (relative to their respective control groups) across days to the same extent, *F*_(10,190)_ < 1.0, *p* > 0.05.

Exposure to noxious stimulation also impaired weight recovery (Figures 2C,D). Relative to pre-treatment (Pre) weight, injury *per se* led to weight loss that slowly recovered over the course of the 4 weeks recovery period, *F*_(10,200)_ = 62.18, *p* < 0.0001. Animals that had received painful stimulation a day after injury recovered less weight. This yielded both a main effect of noxious stimulation, *F*_(1,20)_ = 4.48, *p* = 0.0470, and a Stimulation × Day interaction, *F*_(10,200)_ = 2.74, *p* = 0.0035. Importantly, the effect of noxious stimulation did not vary across pain type, all *F*’s < 1.0, *p* > 0.05.

### Pain Type Affects Acute Cytokine and Caspase Expression

Next, we assessed the acute effect of noxious stimulation on the activation of pro-inflammatory cytokines (TNF, IL-1ß and IL-18) and indices of cell death (caspase-1, 3 and 8). A day after a contusion injury, rats were treated with shock or capsaicin. An equal number of contused rats served as the unshocked or vehicle controls. One cm of tissue encompassing the injury site was collected 1, 3, or 24 h after treatment.

**Figure 3:**
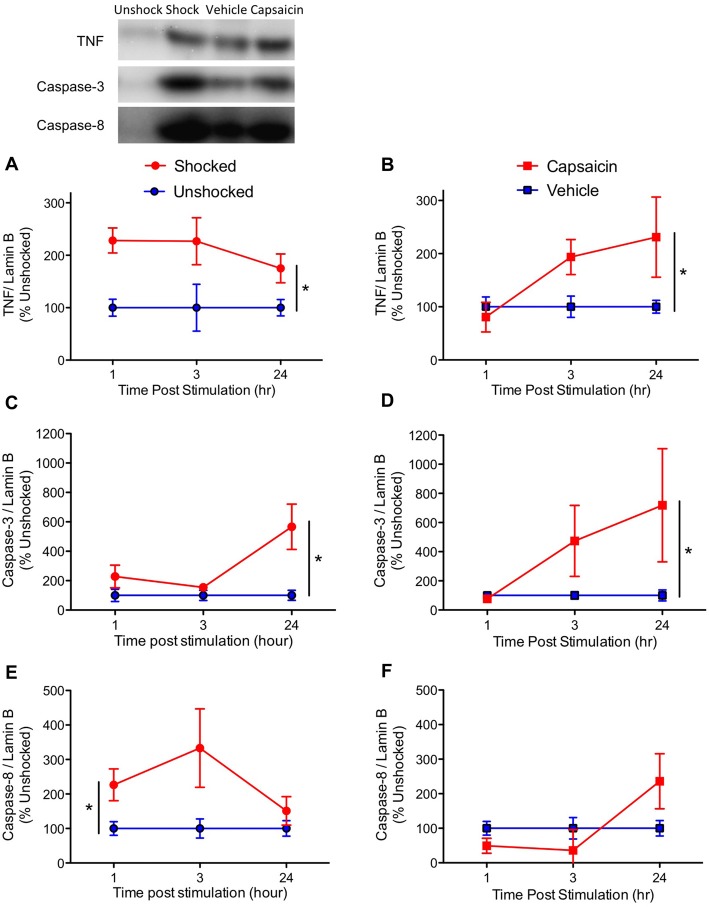
Impact of shock or capsaicin treatment on cell signals associated with apoptosis. Rats exposed to variable intermittent shock (Shocked) a day after injury exhibited greater expression of tumor necrosis factor (TNF) **(A)**, caspase-3 **(C)**, and caspase-8 **(E)**. Capsaicin treatment increased expression of TNF **(B)** and caspase-3 **(D)**, but did not have a significant effect on caspase-8 **(F)**. Representative blots from the 24 h time point are depicted to the right of the figures. Error bars indicate the SEM (*n* = 6). *p < 0.05.

Based on our prior work, we first assessed expression of the inflammatory cytokine TNF, and key mediators of the apoptotic cell death pathway, caspase-3 and 8. Consistent with past results (Huie et al., 2012; Garraway et al., 2014), noxious stimulation increased the expression of TNF (Figures 3A,B), *F*_(1,57)_ = 17.43, *p* < 0.0001. While it appears that TNF expression emerged more slowly after capsaicin treatment, the three-way interaction between Noxious Stimulation, Pain Type and Time did not approach significance, *F*_(2,57)_ = 1.857, *p* > 0.05. Likewise, the main effect of time and pain type, and their interaction, were not statistically significant, *F*’s < 1.0, *p* > 0.05.

Exposure to either shock or capsaicin increased caspase-3 expression (Figures 3C,D), *F*_(1,57)_ = 7.54, *p* = 0.0081. The magnitude of this effect did not depend upon pain type or time, all *F*’s < 2.098, *p* > 0.05.

Noxious stimulation also increased caspase-8 expression (Figures 3E,F), *F*_(1,57)_ = 4.65, *p* = 0.0353. The magnitude of this effect depended upon both pain type and time, with both the Pain Type × Noxious Stimulation, and the Pain Type × Noxious Stimulation × Time interactions approaching statistical significance, both *F*’s > 2.82, *p* < 0.0679. The main effect of pain type also approached significance, *F*_(1,57)_ = 3.76, *p* = 0.0574. To further assess the nature of these effects, additional ANOVAs were performed on the data subdivided by pain type. These analyses revealed that shock had a significant effect, *F*_(1,30)_ = 7.77, *p* = 0.0091, but capsaicin treatment did not, *F*_(2,30)_ < 1.0, *p* > 0.05. No other term approached significance, all *F*’s < 2.01, *p* > 0.05.

**Figure 4:**
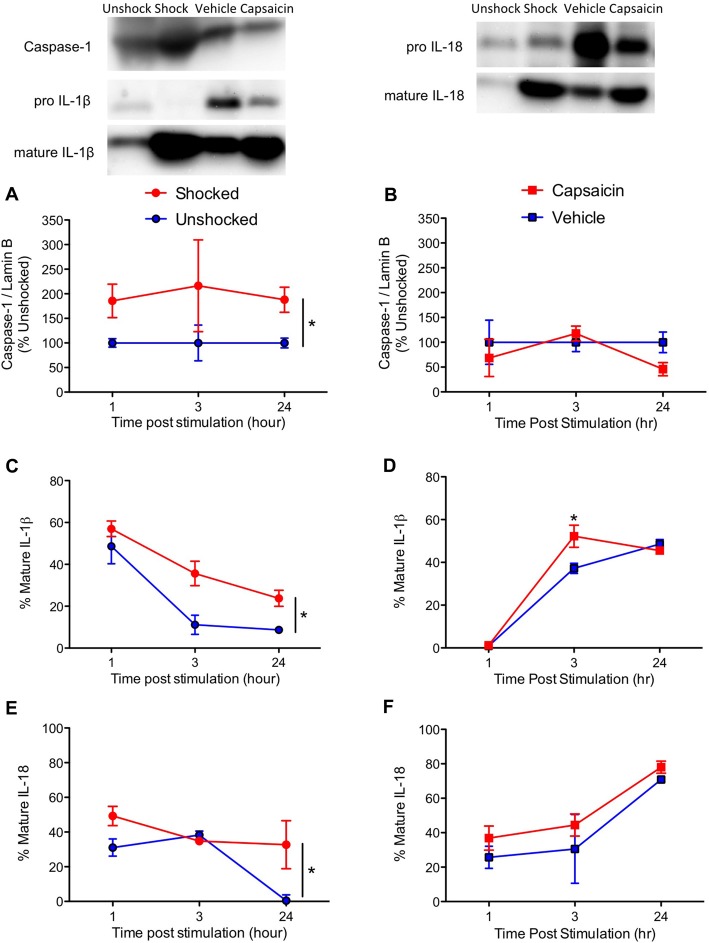
Impact of shock or capsaicin treatment on cell signals associated with pyroptosis. Rats exposed to variable intermittent shock (Shocked) a day after injury exhibited greater expression of caspase-1 **(A)**, IL-1ß **(C)**, and IL-18 **(E)**. Capsaicin treatment had no effect on caspase-1 **(B)**, increased expression of IL-1ß 3 h after treatment **(D)**, and did not have a significant effect on IL-18 **(F)**. Representative blots from 24 h (IL-1ß) after nociceptive stimulation. Error bars indicate the SEM (*n* = 6). *p < 0.05.

We then extended our past observations, focusing on cell signals (caspase-1, IL-1ß and IL-18) related to pyroptotic cell death. Shock exposure increased caspase-1 expression while capsaicin treatment had little effect (Figures 4A,B). This yielded a significant main effect of pain type, *F*_(1,57)_ = 6.17, *p* = 0.160, and a Pain Type by Noxious Stimulation interaction, *F*_(2,57)_ = 6.17, *p* = 0.0160. Separate ANOVAs performed on the data sub-divided by pain type verified that shock had a significant effect, *F*_(1,30)_ = 6.12, *p* = 0.192, while capsaicin treatment did not, *F*_(1,27)_ < 1.0, *p* > 0.5.

IL-1ß expression was enhanced by noxious stimulation (Figures 4C,D), *F*_(1,57)_ = 16.15, *p* = 0.0002, and the magnitude of this effect varied across pain type and time, both *F*’s > 5.67, *p* > 0.05. In addition, the overall levels of IL-1ß expression were higher soon after shock treatment, *F*_(2,57)_ = 3.94, *p* = 0.0250. To further analyze the nature of these interactions, we performed additional ANOVAs with the data sub-divided by pain type. Shock treatment increased IL-1ß expression, *F*_(1,30)_ = 14.16, *p* = 0.0007, and overall levels declined over time, *F*_(2,30)_ = 27.78, *p* = 0.0001. Conversely, vehicle and capsaicin treated animals exhibited a significant increase in IL-1ß expression over time, *F*_(2, 27)_ = 5.76, *p* = 0.0083, and the effect of pain input depended upon time, *F*_(2, 27)_ = 5.76, *p* = 0.0083, with the strongest effect observed 3 h after treatment.

The overall effect of noxious stimulation on IL-18 expression (Figures 4E,F) was not statistically significant, *F*_(1,57)_ = 3.39, *p* = 0.071. While IL-18 expression generally declined over time in the shocked/unshocked groups, it rose after a peripheral injection. This yielded an overall effect of pain type, *F*_(1,57)_ = 5.42, *p* = 0.0234, and a Pain Type by Time interaction, *F*_(2,57)_ = 8.21, *p* = 0.0007. Again, to further analyze the nature of these effects, we performed additional ANOVAs on the data sub-divided by pain type. In both cases, there was a significant effect of time, both *F*’s > 4.34, *p* < 0.05. While shock treatment had an effect, *F*_(1,30)_ = 4.44, *p* = 0.0436, capsaicin treatment did not, *F*_(1,27)_ < 1.0, *p* > 0.05.

**Figure 5:**
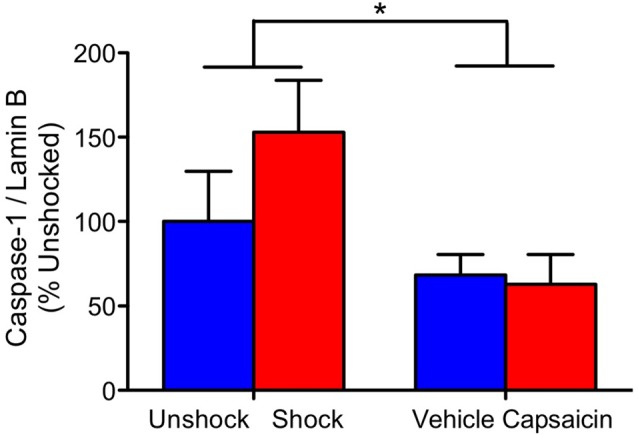
Caspase-1 expression 28 days after contused rats were exposed to nociceptive stimulation. Rats that had previously received an injection (vehicle or capsaicin) exhibited lower caspase-1 expression. Error bars indicate the SEM (*n* = 6). *p < 0.05.

We also examined the expression of pro-inflammatory cytokines and caspase 1, 3, and 8 in rats 4 weeks after treatment. In no case did noxious stimulation have a significant effect, all *F’s* < 1.05, *p* > 0.05. Across all of the analyses, the only significant effect observed stemmed from a general decrease in caspase-1 expression in the previously injected (vehicle and capsaicin) animals (Figure 5), *F*_(1,20)_ = 5.34, *p* = 0.0317.

### BBG Attenuates the Expression of Proteins Related to Pyroptosis

Our results suggest that intermittent electrical stimulation may impair long-term recovery because it fosters pyroptotic cell death. As noted earlier, the activation of caspase-1 is related to the outward flow of ATP through the panexin-1 channel and the activation of P2X7 receptor, which fuels further ATP release (Wicki-Stordeur and Swayne, 2014). This model suggests that blocking either the pannexin-1 channel with probenecid, or the P2X7 receptor with BBG, should attenuate the acute activation of signaling proteins related to pyroptosis. Reasoning that these drugs could have an additive effect, we also assessed the impact of combined (BBG+probenecid) treatment. Drugs were administered starting 3 h after injury, as described above. A day after injury, animals were exposed to shock or nothing (unshocked) and tissue was collected 3 h later. Western blotting was then conducted to assess the activation of ligands linked to apoptotic and pyroptotic cell death.

**Figure 6:**
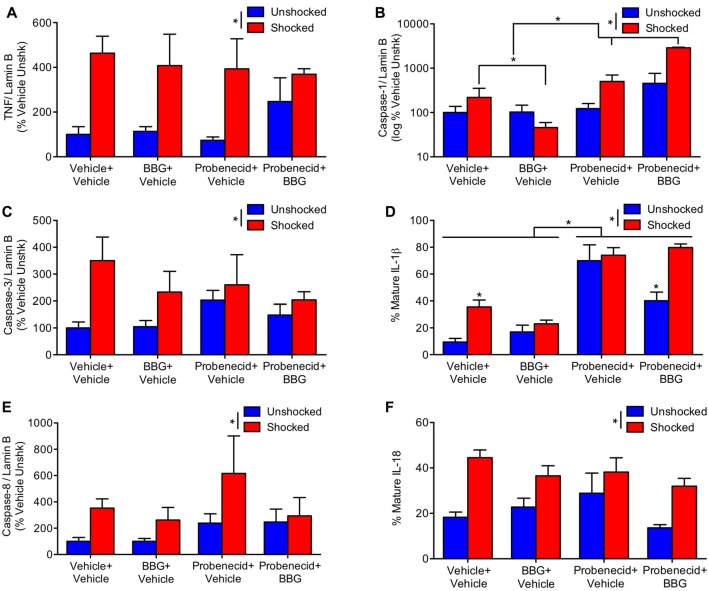
Impact of brilliant blue (BBG) and probenecid on shock-induced protein expression. Contused rats exhibited increased expression of TNF **(A)**, caspase-3 **(C)**, and caspase-8 **(E)** 3 h after they were exposed to shock. BBG attenuated shock-induced caspase-1 **(B)** and IL-1ß **(D)** expression, whereas probenecid generally increased expression. Shock treatment increased IL-18 **(F)** expression. Error bars indicate the SEM (*n* = 6). *p < 0.05.

Shock treatment generally increased the expression of TNF, caspase-3 and caspase-8 (Figures 6A,C,E), all *F*’s > 4.48, *p* < 0.05. While probenecid *per se* appeared to increase TNF, caspase-3 and 8 expression in the unshocked controls, this effect of drug treatment did not approach statistical significance, all *F*’s < 1.84, *p* > 0.05. To further assess the potential effect of drug treatment, *per se*, an additional ANOVA was performed on the data from the unshocked controls. For both caspase-3 and 8, the effect of probenecid approached statistical significance, both *F’s* > 3.78, *p* < 0.0668. For TNF, there was no effect, *F*_(1,19)_ = 1.68, *p* < 0.2105.

Unexpectedly, BBG and probenecid had opposing effects on caspase-1 expression (Figure 6B); whereas BBG attenuated expression, probenecid amplified it. The latter effect was extremely robust (to accommodate the range of values, these data were plotted using a logarithmic scale). An ANOVA confirmed that the main effects of BBG, probenecid, and shock treatment were statistically significant, all *F*’s > 29.86, *p* < 0.0001. So too were all of the higher-order interactions, all *F*’s > 18.08, *p* < 0.0001. To clarify the nature of these interactions, separate ANOVAs were performed on the data from shocked and unshocked rats. For the unshocked animals, drug treatment had no effect, all *F*’s < 1.0, *p* > 0.05. In contrast, in shocked animals there was a main effect of BBG and probenecid treatment, as well as a significant interaction, all *F*’s > 53.33, *p* < 0.0001. This interaction emerged because BBG and probenecid affected caspase-1 expression in opposite ways.

Likewise, BBG reduced IL-1ß expression, while probenecid amplified it (Figure 6D). An ANOVA yielded a main effect of shock and probenecid treatment, both *F*’s > 17.26, *p* < 0.0002. More important, the three-way interaction showed that the effect of shock depended on both BBG and probenecid, *F*_(1, 39)_ = 9.70, *p* = 0.0034. Again, to assess the effect of drug treatment *per se*, we performed an additional analysis on the data from the unshocked animals. This revealed a significant effect of probenecid, *F*_(1,20)_ = 22.44, *p* < 0.0001. Interestingly, the effect of probenecid interacted with BBG treatment, *F*_(1,20)_ = 6.51, *p* = 0. 0190. In shocked animals, BBG and probenecid affected IL-1ß expression in opposite ways. This yielded a main effect of probenecid, *F*_(1,19)_ = 95.32, *p* < 0.0001 and an interaction that approached significance, *F*_(1,19)_ = 3.55, *p* = 0.075. *Post hoc* comparisons showed that shock induced greater expression in the drug-free (vehicle-vehicle) animals and that this effect was amplified by probenecid treatment (*p* < 0.05).

Shock also amplified the expression of IL-18 (Figure 6F), *F*_(1,39)_ = 20.95, *p* < 0.0001. While less IL-18 expression was observed in rats pretreated with BBG, this effect did not reach statistical significance, *F*_(1,39)_ = 3.07, *p* = 0.0874.

### BBG Fosters Long-Term Recovery

Our results suggest that nociceptive stimulation can engage signal pathways linked to pyroptotic cell death and that this acute effect is attenuated by pretreatment with BBG. To evaluate whether pyroptosis contributes to the adverse effect nociceptive stimulation has on long-term recovery, contused rats were pretreated with BBG or its vehicle and exposed to shock or nothing (unshocked) a day after injury. Locomotor recovery was then assessed over the next 6 weeks. In addition, to evaluate whether pyroptosis contributes to the development of chronic pain, mechanical and thermal reactivity was assessed at the end of the recovery period.

**Figure 7:**
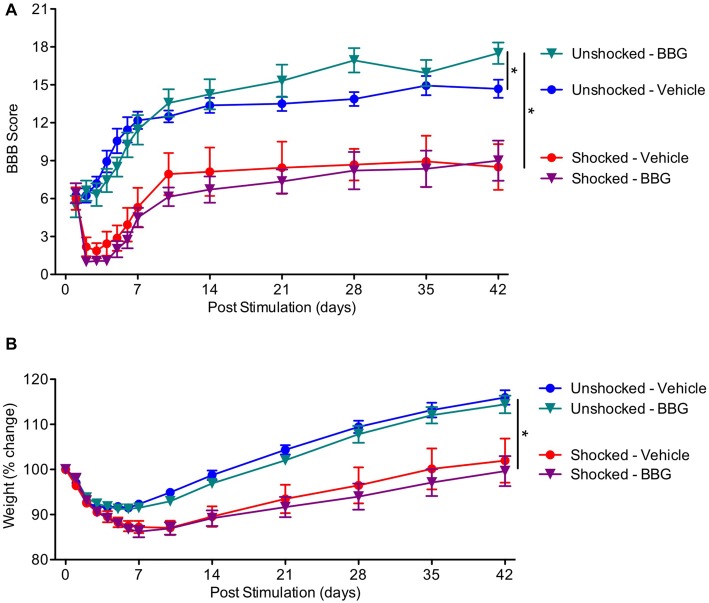
Impact of BBG on recovery in animals exposed to shock. **(A)** Pretreatment with BBG enhanced locomotor recovery in unshocked animals, but had no effect on rats that had received shock. **(B)** Shock treatment undermined the recovery of weight. Error bars indicate the SEM (*n* = 6). *p < 0.05.

As expected, locomotor performance improved over the course of the recovery period (Figure 7A), *F*_(13,351)_ = 8.40, *p* < 0.0001. Exposure to shock impaired long-term recovery, and the magnitude of this effect became stronger across days, both *F*’s < 2.53, *p* < 0.05. Over days, BBG treatment improved performance in the unshocked, but not the shocked rats, yielding significant Day × BBG and Day × BBG × Shock treatment interactions. To assess the nature of the three-way interaction, additional ANCOVAs were perform on the unshocked and shocked groups. In the unshocked rats, there was an effect of BBG treatment that became stronger across days, *F*_(11,143)_ = 6.23, *p* = 0.0001. BBG treatment had no effect on locomotor recovery in rats that had received shock, both* F*’s < 1.0, *p* > 0.05.

Shock treatment also slowed the rate at which animals recovered weight over days (Figure 7B). An ANOVA performed on the % change yielded a main effect of shock treatment, *F*_(1,27)_ = 26.03, *p* < 0.0001, and a Day × Shock treatment interaction, *F*_(11, 297)_ = 17.08, *p* = 0.0001. There was no effect of BBG treatment, all *F*’s < 1.0, *p* > 0.05.

**Figure 8:**
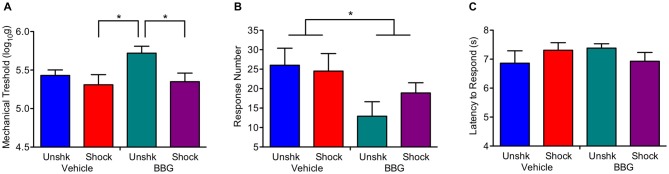
Impact of BBG and shock treatment on nociceptive reactivity at the end of the recovery period. **(A)** Shock treatment lowered the threshold for eliciting a response to mechanical stimulation of the paw. **(B)** Pretreatment with BBG reduced the number of vocalizations elicited by mechanical stimulation to the girdle region. **(C)** Neither shock nor BBG treatment affected reactivity to a thermal stimulus applied to the tail. Error bars indicate the SEM (*n* = 6). *p < 0.05.

At the end of the recovery period, shocked rats were generally more responsive to mechanical stimulation applied to the hind paw (Figure 8A), *F*_(1,27)_ = 5.58, *p* = 0.0257. Neither the main effect of BBG treatment, *F*_(1,27)_ = 2.82, *p* > 0.05, nor its interaction with shock treatment, *F*_(1,27)_ = 1.50, *p* > 0.05, were statistically significant. Shock treatment had no effect on behavioral reactivity on the girdle test (Figure 8B), both *F*_(1,26)_ < 1.0. *p* > 0.05. Independent of shock treatment, BBG reduced the number of vocalizations elicited during stimulation of the girdle region, *F*_(1,26)_ = 5.70, *p* = 0.0245. Neither BBG nor shock treatment affected responsiveness to a thermal stimulus applied to the tail (Figure 8C), all *F*’s < 2.27, *p* > 0.05.

## Discussion

Prior work has shown that nociceptive stimulation impairs adaptive plasticity caudal to a complete spinal transection, an effect that has been linked to the activation of C-fibers and the sensitization of nociceptive processes within the lumbosacral spinal cord (Grau et al., 2006, 2012; Baumbauer et al., 2008; Hook et al., 2008; Ferguson et al., 2012). Supporting this, we have shown that intermittent shock only impairs learning if it is given at an intensity that engages C-fibers and that chemically activating TRPV1 expressing C-fibers with capsaicin has the same effect (Baumbauer et al., 2008; Hook et al., 2008). We had previously extended this work to a contusion injury, demonstrating that just 6 min of intermittent electrical stimulation a day after injury increases tissue loss and impairs long-term recovery (Grau et al., 2004). This effect has been linked to the activation of TNF and signals (caspase-3 and 8) related to the initiation of cell death (Garraway et al., 2014). The present article explored the generality of this effect and whether nociceptive stimulation engages signals linked to pyroptotic cell death.

We examined the generality of the phenomenon by testing the effect of the irritant capsaicin. To evaluate whether capsaicin and shock engage a comparable level of neural excitation at the site of injury, we assessed *c-fos* mRNA expression (Gao and Ji, 2009). Six minutes of intermittent shock applied to the tail and an intradermal injection of 3% capsaicin applied to one hind paw engaged *c-fos* to roughly the same extent. We then compared the impact of these treatments on long-term recovery. Both undermined the recovery of locomotor function and enhanced weight loss. Interestingly, rats that received an intradermal injection exhibited poor recovery relative to the rats that had been just restrained for a comparable period of time (unshocked). Because an intradermal injection induces some local irritation/swelling, this observation is consistent with our finding that nociceptive input after injury can impair long-term recovery (Grau et al., 2004, 2012). What is surprising is that this relatively subtle nociceptive input had an effect. The finding suggests that minor clinical interventions (e.g., laceration repair or debridement) after injury could have an unintended adverse effect.

To explore the acute effect of nociceptive stimulation, rats were exposed to shock or capsaicin a day after injury and tissue was collected 1, 3, or 24 h later. As previously reported (Garraway et al., 2014), we found that intermittent shock increased the expression of TNF, caspase-3 and 8. Treatment with capsaicin had a comparable effect. These finding imply that both forms of noxious stimulation engage signal pathways that promote apoptotic cell death.

Exposure to noxious electrical stimulation also enhanced the expression of ligands linked to pyroptotic cell death (caspase-1, IL-1ß and IL-18; Fink and Cookson, 2005; Bergsbaken et al., 2009; Duprez et al., 2009). Treatment with capsaicin had relatively little effect, producing an increase in IL-1ß that was statistically significant at just one time point (at 3 h). Interestingly, the overall pattern of IL-1ß and IL-18 expression differed across treatment conditions, an effect that was particularly evident from a comparison of the unshocked and vehicle treated controls. Unshocked animals exhibited a general decline in IL-1ß and IL-18 expression from 1 h to 24 h after noxious stimulation (Figure 4). In contrast, rats that received an intradermal injection of the vehicle exhibited an increase in expression over time. The latter observation again suggests that the injection *per se* had an effect, promoting the development of inflammation at the site of injury.

Assays conducted on the tissue collected 4 weeks after injury found no effect of nociceptive stimulation. The only statistically significant finding was related to a general reduction in caspase-1 expression in animals that had previously received an intradermal injection.

Our results imply that both intermittent shock and the application of a peripheral irritant foster cell death after injury and impair long-term recovery. However, the cellular response to stimulation varied with pain type, impacting the degree to which alternative forms of cell death were engaged. It appears that a stimulus that tonically engages just C-fibers (capsaicin) fosters apoptotic cell death. In contrast, intermittent electrical stimulation, which activates a broad range of sensory fibers in a phasic manner, amplified the expression of signal pathways related to both apoptosis and pyroptosis. The implication is that how pain input and polytrauma affect the development of secondary injury may vary depending upon the nature of the tissue damage/stimulation.

We then examined whether drug treatments that target pyroptosis impact the adverse effect of shock treatment. The development of pyroptosis has been linked to the release of ATP through the pannexin-1 channel, which is regulated by the protein receptor P2X7 (Dahl and Keane, 2012; de Rivero Vaccari et al., 2014, 2016). Extracellular ATP can engage the P2X7 receptor, which further engages the pannexin-1 channel, fueling the release of ATP and enlarging the channel beyond its normal functional bounds. This allows cytokines to flow out of the cell, spreading the inflammatory fire, and the inward flow of extracellular molecules (e.g., Ca^++^) that promote cell death. To inhibit these processes, we administered the P2X7 receptor antagonist BBG and/or the pannexin-1 channel inhibitor probenecid. Neither drug treatment had a significant effect on the shock-induced expression of TNF or ligands related to apoptosis (caspase-3 and 8). Administration of BBG prior to shock reduced the expression of caspase-1 and IL-1ß. This effect was not observed after probenecid. Indeed, probenecid treatment *per se* generally increased the expression of caspase-1 and IL-1ß. Interestingly, the enhancement in IL-1ß expression observed in the probenecid treated unshocked animals was attenuated by co-administration of BBG.

The fact BBG attenuates caspase-1 and IL-1ß expression supports the framework outlined above, wherein the activation of the P2X7 receptor fuels the development of pyroptosis, and suggests that this drug treatment may have therapeutic value (Peng et al., 2009; de Rivero Vaccari et al., 2014, 2016). The effect of probenecid, however, appears to run counter to the standard view, which implied that inhibiting the pannexin-1 channel should dampen pyroptosis. At a functional level, it seems that the administration of probenecid engaged a compensatory process that amplified the activation of caspase-1 and IL-1ß. It is not clear why this occurred. The observation does, however, fit with the relative paucity of data demonstrating a therapeutic effect of probenecid on recovery, relative to BBG.

The last experiment assessed whether pretreatment with BBG attenuates the adverse effect of shock on long-term recovery. We again found that shocked animals exhibit poor locomotor recovery and less regain of weight. Pretreatment with BBG had no effect on these measures. Interestingly, BBG did promote locomotor recovery in the unshocked controls, replicating past work (Peng et al., 2009; but also see Marcillo et al., 2012). Also, as previously reported (Garraway et al., 2014), shock treatment enhanced reactivity to mechanical stimulation applied to the paw. This effect was not affected by drug treatment. BBG did, however, generally reduce responsiveness to mechanical stimulation applied to the girdle region, implying that the drug may attenuate the development of chronic pain.

The fact that BBG does not reduce the adverse effect of shock on recovery implies that pyroptosis may reflect a down-stream consequence of another process that sets the stage for cell death. Recent work suggests that this process is related to the breakdown of the blood spinal cord barrier (BSCB). Supporting this, Turtle et al. (2017) showed that the application of shock or capsaicin a day after rats received a contusion injury increased the infiltration of red blood cells into the injured tissue. Because hemoglobin is cytotoxic (Regan and Guo, 1998), this would fuel cell death and the expansion of injury. We have related these effects to the up-regulation of Sur1-Trpm4 channel (Grau et al., 2017), which can fuel the loss of endothelial cells and lead to capillary fragmentation through a process known as progressive hemorrhagic necrosis (Simard et al., 2007, 2012).

Our findings suggest that nociceptive input after injury can expand the area of injury and impair long-term recovery. We have related these effects to the activation of unmyelinated pain (C) fibers. It is important to note, however, that these processes are tied to pain-related nociceptive activity, not to psychological (brain-dependent) pain. This conclusion is based on work examining the effect of systemic morphine, given at a dose that completely blocks both spinal and brain-dependent responses to nociceptive stimulation (Hook et al., 2007). Morphine had no effect on shock-induced hemorrhage (Turtle et al., 2017). Nor did it attenuate the effect of shock treatment on long-term recovery (Hook et al., 2007). Indeed, morphine *per se* had an adverse effect on recovery (Hook et al., 2007, 2009), an observation that suggests caution regarding its use in the acute treatment of pain after injury. Interestingly, the adverse effect of morphine on recovery is related to an up-regulation of IL-1ß (Hook et al., 2011), which suggests opiate treatment may foster pyroptotic cell death.

More recently Turtle et al. (2017) tested the effect of inhibiting cellular activity using the Na^+^ channel blocker lidocaine. Contused rats were given lidocaine prior to shock treatment a day after injury. We found that lidocaine attenuated shock-induced hemorrhage. More importantly, lidocaine completely blocked the adverse effect nociceptive stimulation has on long-term recovery, implying that epidural lidocaine could have therapeutic value in cases of polytrauma.

## Author Contributions

The study was conducted by JT in partial fulfillment of the degree requirements for a Ph.D. in Neuroscience at Texas A&M University. JT orchestrated the performance of the work, suggested pyroptosis could play a role, and wrote the first draft of this manuscript. MS, SG and JG provided input on pain processes, TNF and apoptosis. The data were collected by JT, MS, JR, Y-JH, KL and MH. The results were analyzed by JT and JG. The article was written by JT and JG, with input from MS, JR, Y-JH, KL, MH and SG.

## Conflict of Interest Statement

The authors declare that the research was conducted in the absence of any commercial or financial relationships that could be construed as a potential conflict of interest.
